# Pea p68, a DEAD-Box Helicase, Provides Salinity Stress Tolerance in Transgenic Tobacco by Reducing Oxidative Stress and Improving Photosynthesis Machinery

**DOI:** 10.1371/journal.pone.0098287

**Published:** 2014-05-30

**Authors:** Narendra Tuteja, Mst. Sufara Akhter Banu, Kazi Md. Kamrul Huda, Sarvajeet Singh Gill, Parul Jain, Xuan Hoi Pham, Renu Tuteja

**Affiliations:** 1 International Centre for Genetic Engineering and Biotechnology, Aruna Asaf Ali Marg, New Delhi, India; 2 Stress Physiology and Molecular Biology Lab, Centre for Biotechnology, MD University, Rohtak, India; Institute of Genetics and Developmental Biology, Chinese Academy of Sciences, China

## Abstract

**Background:**

The DEAD-box helicases are required mostly in all aspects of RNA and DNA metabolism and they play a significant role in various abiotic stresses, including salinity. The *p68* is an important member of the DEAD-box proteins family and, in animal system, it is involved in RNA metabolism including pre-RNA processing and splicing. In plant system, it has not been well characterized. Here we report the cloning and characterization of *p68* from pea (*Pisum sativum*) and its novel function in salinity stress tolerance in plant.

**Results:**

The pea p68 protein self-interacts and is localized in the cytosol as well as the surrounding of cell nucleus. The transcript of pea *p68* is upregulated in response to high salinity stress in pea. Overexpression of *p68* driven by constitutive cauliflower mosaic virus-35S promoter in tobacco transgenic plants confers enhanced tolerances to salinity stress by improving the growth, photosynthesis and antioxidant machinery. Under stress treatment, pea *p68* overexpressing tobacco accumulated higher K^+^ and lower Na^+^ level than the wild-type plants. Reactive oxygen species (ROS) accumulation was remarkably regulated by the overexpression of pea *p68* under salinity stress conditions, as shown from TBARS content, electrolyte leakage, hydrogen peroxide accumulation and 8-OHdG content and antioxidant enzyme activities.

**Conclusions:**

To the best of our knowledge this is the first direct report, which provides the novel function of pea *p68* helicase in salinity stress tolerance. The results suggest that p68 can also be exploited for engineering abiotic stress tolerance in crop plants of economic importance.

## Introduction

The DEAD-box families of proteins are helicases conserved from bacteria to humans and are involved in a variety of nucleic acid metabolic processes such as replication, repair, recombination, transcription, pre-mRNA processing, RNA degradation, RNA export, ribosome assembly and translation [1]–[3]. At the sequence level, helicases have been classified into five superfamilies (SF1–SF5). The largest of these groups are SF1 and SF2. Most of these contain nine conserved helicase motifs, Q, I, Ia, Ib, II, III, IV, V and VI. All the helicases exhibit nucleic acid dependent ATPase activity which provides energy for the helicase action [3], [4].

The p68 is a prototype member of DEAD-box family and is one of the best characterized helicases and it plays a very important role in cell/organ development and participates in a variety of biological processes including pre-mRNA and pre-rRNA processing [5]–[6], rearrangement of RNA secondary structures [6], RNA splicing [7]–[8] and gene transcription. In human, *p68* is a RNA-binding protein endowed with an ATP-dependent RNA helicase, RNA-dependent ATPase and RNA-protein complex remodeling activities. In human malaria parasite *Plasmodium falciparum,* p68 has also been reported as a dual helicase and its helicase and ATPase activities are stimulated after phosphorylation with protein kinase C [9]–[10].

The DEAD-box RNA helicases are becoming a subject of attention as they play a significant role during development and stress responses in plants [11]–[14]. Each DEAD box RNA helicase is thought to be differentially regulated during development and in response to environmental stresses in plants [2], [13]. *Arabidopsis* LOS4 and RCF1 (a DEAD-box RNA helicase) were reported to regulate gene expression in response to chilling stress [8], [15]. The overexpression of pea DNA helicase (PDH45) was shown to confer salt tolerance in tobacco and rice [12], [14]. A rice DEAD-box RNA helicase (OsBIRH1) has been reported to function in defense responses against pathogen and oxidative stresses [16]. OsSUV3, a member of DEAD-box RNA helicase, has been shown to be involved in salinity stress tolerance recently [17]. Splicing factors are also known to affect the alternative splicing of many genes which lead to alter the gene expression through splicing factor networks and thereby involving in plant stress tolerance [18]. The plant homologue of animal splicing factor p68 has not been characterized in detail till date. The transcript of *Arabibopsis thaliana p68* DEAD-box RNA helicase (AtDRH1) was reported to be accumulated at a high level and almost equally in every part of the *Arabidopsis* plant [19]. Studies on MA16 (maize RNA-binding protein) and ZmDRH1 (*Z. mays* DEAD-box RNA helicase 1) revealed that these proteins might be part of a ribonucleoproteins complex involved in ribosomal RNA (rRNA) metabolism [20]. However, the precise role of DEAD-box RNA helicase, especially the role of *p68* in abiotic stress tolerance, has not been reported so far. Here, we report detailed characterization of *p68* from pea (*Pisum sativum*). Our results show that pea p68 self-interacts and the overexpression of pea p68 enhances the salinity stress tolerance in tobacco by controlling the generation of stress-induced reactive oxygen species (ROS) through modulating antioxidative defence machinery thereby protecting the photosynthesis and yield.

## Materials and Methods

### Construction of Plasmid for Tobacco Transformation

The *pea p68* gene was isolated by screening of pea cDNA library using a heterologous probe from *Arabidopsis thaliana.* The complete ORF of *p68* was cloned into pGEMT easy vector and sequenced (Accession number: AF271892.1). The insert (1.8 kb) was release from pGEMT-*p68* by digesting with EcoRI and BamHI restriction enzymes and ligated into the MCS of pRT101. The CaMV35S-*p68*-polyA cassette generated in pRT101 was then cut with Hind III and ligated to the MCS of pCAMBIA-1301 binary vector containing GUS as the reporter and hygromycin phosphotransferase as the selection marker gene. The pCAMBIA1301-*p68* clone was then transformed into *Agrobacterium* LBA4404 strain for generation of transgenic tobacco using standard protocol [21]. Positive transgenic lines were confirmed by PCR using gene specific primers (FP: 5′- **GAATTC**ATGTCGTATGTTCCTCCACAC-3/EcoRI site bold) and (RP: 5′- **GGATCC**CCATTACCTACAAACATGACTGAT-3/BamHI site bold). The transgenic tobacco plants were analyzed in two independent experiments with three technical replicates.

### Transcript Analysis and Yeast-two-hybrid Assay

To analyze the expression of pea *p68* transcripts, northern blots analysis was performed as described earlier [11]. The pGBKT7-*p68* and pGADT7-*p68* were generated by inserting a PCR fragment encoding the complete ORF of *p68*. The empty yeast strain AH109, pGADT7, pGBKT7, pGADT7/pGBKT7, pGADT7/pGBKT7-*p68*, pGADT7-*p68*/pGBKT7 and pGBKT7-*p68*/pGADT7-*p68* were transformed separately on yeast cells. Yeast cells carrying all the plasmids were selected on the synthetic medium lacking Leu and Trp (SD-Leu-Trp-). The yeast cells were then streaked on SD medium [(Leu), (Trp), (His)] containing 30 mM 3-AT (3-Amino-1, 2, 4-triazole) to determine the expression of HIS3 nutritional reporter. The β-galactosidase expression of the fusion proteins encoded by pGBKT7-*p68*, pGADT7-*p68* constructs was assayed by colony filter lift assays as per manufacturer instruction (Clontech).

### *In vivo* Localization by Immunofluorescence Staining and Confocal Microscopy

Polyclonal antibody against pea p68 was raised in rabbit as per the standard procedure. For *in vivo* localization, exponentially growing tobacco BY2 [11] suspension cells were fixed in 4% formaldehyde, permeabilized by cellulase and layered onto poly-L-lysine-coated cover slips. The cells were immunostained with p68-specific primary rabbit antibody in 1∶2000 dilutions and Alexafluor 488-labeled goat antirabbit secondary antibody (Molecular Probes, Eugene, OR, USA) in 1∶1000 dilutions. Counter staining of the cells with DAPI (4/, 6-diamidino-2-phenylindole), confocal laser scanning microscopy and image processing was carried out in a method described earlier [11].

### Southern and Western Blots Analysis

Genomic DNA was isolated by CTAB method from PCR positive *p68* tobacco transgenic lines and WT. For southern blots analysis, ∼20 µg of genomic DNA was digested with HindIII and resolved on agarose gel. The DNA was transferred to nylon membrane (Hybond N, Amersham Pharmacia, http://www.gelifesciences.com/), and hybridized with radiolabelled *p68* cDNA as described previously [12]. For western blot analysis, the total soluble proteins were isolated from the unstressed tissue samples of transgenic lines as well as WT plants and separated on 12% SDS-PAGE. Western blot analysis was performed by using anti-*p68* (1∶5,000 dilutions) as primary and anti-rabbit (alkaline phosphatase conjugated antirabbit antibody-Sigma) as secondary antibody (1∶12,500 dilutions). The blot was developed as per manufacturer’s protocol (Sigma, USA).

### Histochemical GUS Staining and Morphological Characterization of Transgenic Plants

The seedlings of pea *p68* overexpressing tobacco transgenic lines and WT were vacuum infiltrated for 10 m and histochemical *GUS* staining was performed by a method described earlier [22]. For *in vitro* pollen germination, mature pollen was isolated aseptically and cultured on pollen germination media (SMM: 0.3 M sucrose, 1.6 mM H_3_BO_3_, 3 mM Ca(NO_3_)_2_ 4H_2_O, 0.8 mM MgSO_4_.7H_2_O, 1 mM KNO_3_) supplemented with 100 and 200 mM NaCl and incubated at 26°C in the dark. The germination status of the pollens was monitored and scored during a 7 d experimentation period. The sensitivity of seed germination to NaCl was assayed on MS agar plates saturated with 200 mM NaCl and incubated at 26°C under cool-white light for germination. Leaf disks assays, measurement of growth characteristics like shoot length, root length, leaf area and plant dry weight, measurement of tolerance index, determination of the total chlorophyll content and yield characteristics of transgenic and wild-type (WT) plants were performed as described earlier [14].

### Measurement of Photosynthetic Characteristics and Photosystem II Activity (*F_v_/F_m_*)

The photosynthetic characteristics like net photosynthetic rate (P_N_), stomatal conductance (gs), intercellular CO_2_ concentration (C*i*) and chlorophyll fluorescence (*F_v_/F_m_*) of transgenic lines and WT plants were recorded on the fully expanded leaves using infra*–*red gas analyzer (Li*–*6400, Li*–*COR, Lincoln, NE, USA) between 11∶00 and 12∶00 h. The conditions during the measurement were photosynthetically active radiation (PAR) 945±8 µmol m*^–^* ^2^ s*^–^* ^1^, relative humidity 75±6%, temperature 28±2°C and an ambient CO_2_ concentration of 350 µmol mol*^–^* ^1^. The chlorophyll fluorescence i.e. maximal efficiency of PSII photochemistry (*F_v_/F_m_*) was also determined on the same leaves used for photosynthetic measurements after dark adaptation for 30 min.

### Measurement of Ion Content

The Na^+^ and K^+^ ion content was measured as described earlier [23]. Salinity treated (0, 100 or 200 mM NaCl) leaves of the transgenic lines and WT plants were collected and rinsed with deionised water thoroughly. The fresh weight was determined for each sample. After drying (70°C for 48 h), dry weight was also measured. The samples are then subjected to an overnight digestion with HNO_3_/H_2_O_2_. The materials were picked in 2 M HCl, and Na^+^ and K^+^ ion content was analyzed by using simultaneous inductively coupled plasma emission spectrometry (ICP trace analyzer, Labtam, Braeside, Australia).

### Measurement of Oxidative Stress, Enzymatic Antioxidants (SOD, CAT, APX, GR) and Non-enzymatic Antioxidants (AsA and GSH) in *p68* Transgenic Lines and WT

Oxidative stress was detected by measuring thiobarbituric acid reactive substances (TBARS), hydrogen peroxide (H_2_O_2_) content, electrolyte leakage and oxidative DNA damage (8-OHdG) in the leaves of *p68* overexpressing transgenic lines and WT plants by a previously described method [14]. The salinity stressed (0, 100 or 200 mM NaCl) and fully expanded leaves from transgenic and WT plants were used for the measurement of enzymatic antioxidants (superoxide dismutase, catalase, ascorbate peroxidase, glutathione reductase) and non-enzymatic antioxidants (ascorbate and glutathione). Leaf samples (transgenic lines and WT) were homogenized with an extraction buffer containing 100 mM potassium phosphate buffer (pH 7.0), 0.5% Triton X*–*100 and 1% polyvinylpyrrolidone (PVP) using pre-chilled mortar and pestle. The homogenate was centrifuged at 15,000×g for 20 min at 4°C. The supernatant obtained after centrifugation was used for enzyme assays. Measurement of enzymatic antioxidants (SOD, CAT, APX, and GR) and non-enzymatic antioxidants (AsA and GSH) was performed as described previously [14]. All the measurements were carried out 3 weeks after initiating the NaCl treatment.

### Transgenic Plants and Salinity Stress Tolerance

For the assay of sensitivity to salinity stress, 14 d-old seedlings of WT and the transgenic plants (grown on vermiculite pots) were transferred to nutrient solutions containing 200 mM NaCl. After 2 d, growth status of the transgenic lines was observed. In another experiment, 40 plants of each line and WT were grown in vermiculite pots and watered for 10 d and then 200 mM NaCl solution was irrigated for every 3 d interval up to 12 d. After treatments, morphological changes were observed. In order to investigate the effect of salinity stress, 35-day-old plants were also subjected to salt stress for 28 d and growth was observed till maturity and photographs were taken.

### Statistical Analysis

All the treatments were performed in three independent trials with consistent results. The results from only one representative experiment are shown, expressed as means± standard errors. Analysis of one-way variance (ANOVA) was performed on the data using SPSS (12.0 Inc., USA) to determine the least significant difference (LSD) for the significant data to identify the differences in the mean among the treatments. The means were separated by Duncan’s multiple range tests (DMRT). The graphs were prepared using Sigmaplot Ver. 11. Different letters indicate significant difference at *P<0.05*.

## Results

### Isolation and Sequence Analysis of Pea p68 cDNA

The pea cDNA library was screened using a 1.9 kb cDNA fragment of *p68* from *Arabidopsis thaliana* (kindly provided by Tetsuo Meshi of Kyoto University, Japan) as a probe. This resulted in the isolation of a positive clone of pea *p68* (Figure S1A). The sequence analysis shows that the open reading frame (ORF) of pea *p68* cDNA (1.8 kb) encodes a protein of 622 amino acid residues, with a calculated molecular mass of 67.65 kDa and a pI of 6.46. It also exhibits all the known canonical helicase motifs (Q, I, Ia, Ib, II-VI) (Figure S1B). Phylogenetic analysis identified the closest orthologs of pea *p68* as *Arabidopsis thaliana* and *Saccharomyces cerevisiae p68* followed by *Oryza sativa p68* (Figure S1C).

### Tissue Specific Distribution and Regulation of Pea *p68* Transcript in Response to Abiotic Stresses

**Figure 1:**
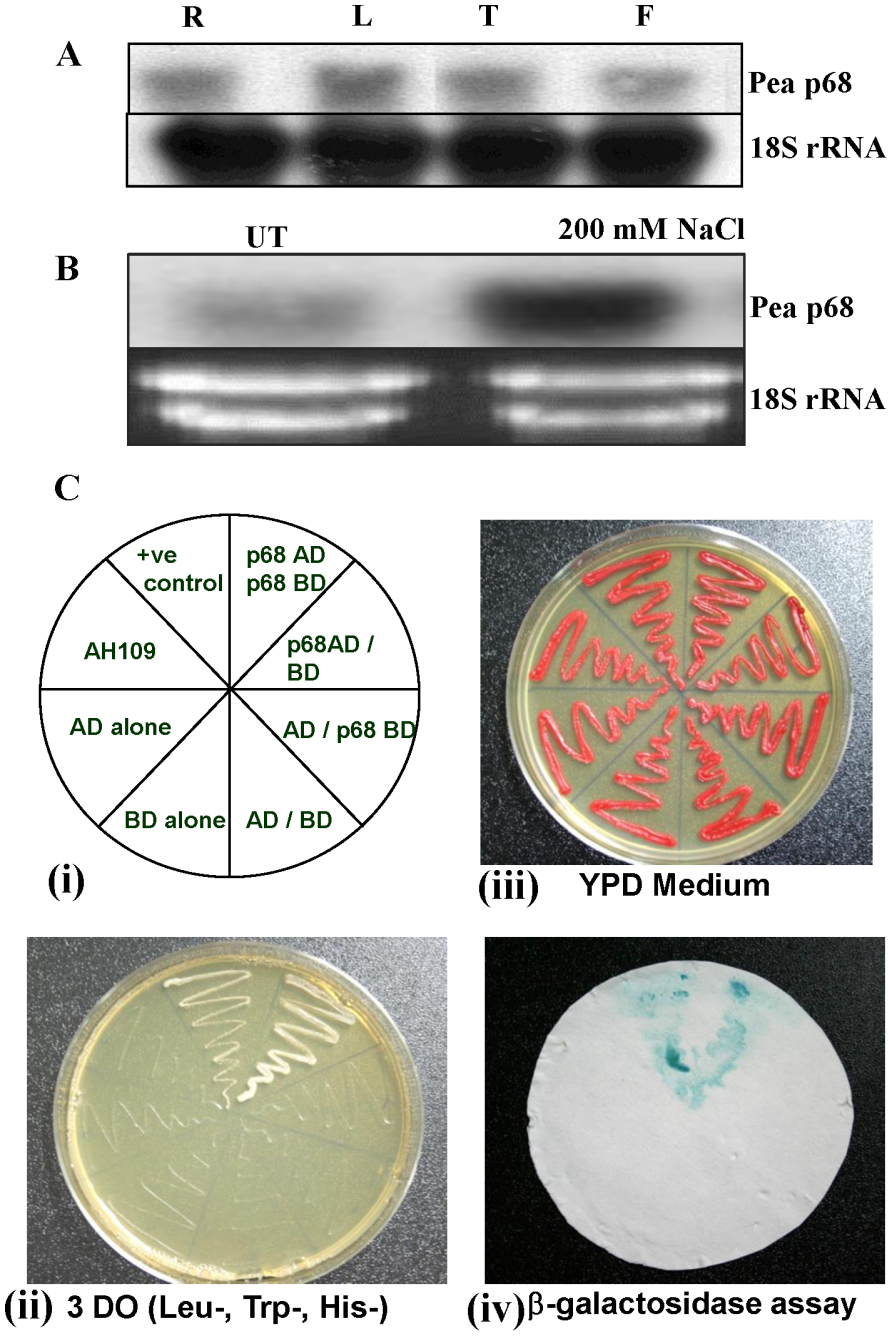
Expression analysis and self-interaction assay for pea *p68*. (A). Transcript analysis of pea p68 by Northern blot analysis (R: root, S: shoot, T: tendril and F: flower tissue respectively). (B) Transcript level in response 200 mM NaCl stress respectively (UT: untreated samples). About 30 µg of total was separated by electrophoresis, blotted and hybridized with the ^32P^-labeled ORF of pea p68 cDNA (1.8 kb). For equal loading of RNA in each lane, the same blot was hybridized with the 18S rRNA, as shown in bottom of the each panel. (C) Yeast two-hybrid system-based self interaction showing pea p68 interacts with pea p68 *in vivo* (i) Template showing the organization of Y2H experiment (ii) Showing phenotypes on a YPD plate (iii) Yeast growing on a synthetic dextrose plate lacking leucine, tryptophan and histidine (3 DO) and (iv) β-galactosidase filter lift assay showing positive pea p68 self interaction. Yeast strain (AH109) carrying and *Gβ*-AD+*Gγ*-BD used as a positive control for yeast two-hybrid assay.

To investigate the expression pattern of the transcript level of pea *p68* in different parts of pea plant, the total RNA was isolated from root, leaf, tendril and flower and was subjected to northern blot analysis using 1.8 kb pea *p68* cDNA as the probe. For the internal control, 18S ribosome probe was used to show the equal loading. A single transcript of the expected size for pea *p68* mRNAs was present in all the tissues (Figure 1A). Further, to analyze the expression of pea *p68* under salinity stress, 7-day old pea seedlings were treated with 200 mM NaCl for 24 h and used for northern blot analysis. The results show that the pea *p68* transcript was dramatically up-regulated (∼4.8 fold) in response to salinity stress (Figure 1B).

### Pea *p68* Interacts with Itself in the Yeast Two-hybrid System

To verify the self-interaction, we co-expressed BD-pea p68 with a construct encoding the full ORF of pea *p68* fused to the GAL4 activation domain (AD-Pea p68). Yeast cells carrying both the plasmids of BD-pea p68+ AD-Pea p68 were able to grow on 3DO media [Figure 1C (ii)]. All the clones including negative and positive control transformed in yeast cells were grown in YPD medium [Figure 1C (iii)]. Yeast cell carrying the plasmids of BD-pea p68+ AD-Pea p68 and Gβ-pGADT7+Gβ-pGBKT7 exhibited β-galactosidase activity, indicating the expression of reporter genes [Figure 1C (iv)]. These results confirm that the full-length pea p68 is able to self-interact in the yeast two-hybrid system.

### *In vivo* Localization of p68

**Figure 2:**
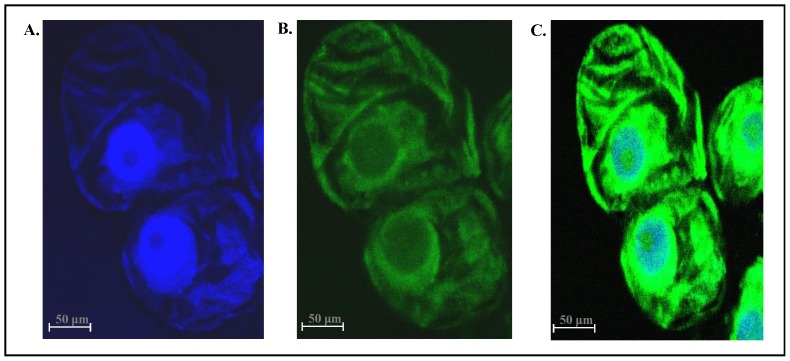
*In vivo* localization of the p68 in tobacco BY2 cells. The tobacco BY2 cells were fixed, permeabilized and immunostained with primary antibodies against p68 followed by Alexafluor 488-labeled secondary antibody and then counterstained with DAPI. A single confocal image is shown. (A) Image of cell stained with DAPI (blue). (B) Immunofluorescently stained cell (green). Anti-p68 labeling is restricted to the nucleus and cytosol. (C) Superimposed image of cell.

The *in vivo* localization of p68 was also analyzed by immunofluorescence labeling and observed under confocal microscopy. Immunofluorescent labeling of tobacco BY2 cells with anti-pea p68 antibodies showed p68 protein exclusively localized in the cytoplasm as well as in the surrounding of cell nucleus (Figure 2).

### Overexpression of *p68* and Molecular Analysis of Transgenic Tobacco Lines

**Figure 3:**
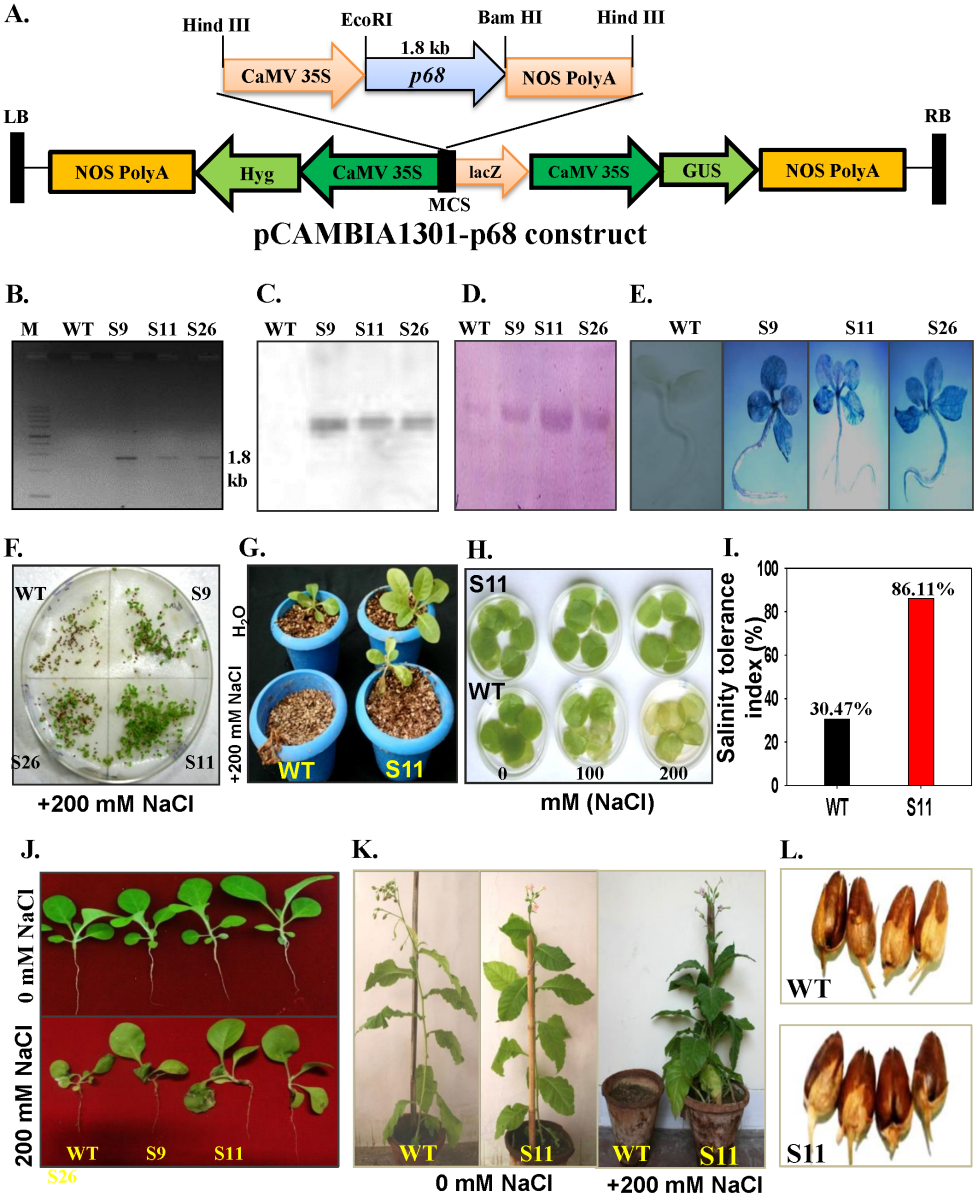
Molecular and morphophysiological analysis of pea *p68* overexpressing transgenic tobacco plants. (A) The map of the construct of pCAMBIA1301 containing the *p68* gene (pCAMBIA1301-*p68*). (B) PCR analysis using gene-specific primers. (C) Southern blot analysis for the integration of pea *p68* gene in tobacco genome. (D) Western blot analysis of each transgenic lines using anti-pea *p68* polyclonal antibody. (E) Histochemical GUS staining of each transgenic line. (F) Comparison of seeds germination of the WT and transgenic lines in response to 200 mM NaCl. (G) Phenotypic comparison of WT and transgenic line (S11) in response to 200 mM NaCl stress. 21 d-old-seedling growing in vermiculite pots and supplied with 200 mM NaCl solution for 5 d. (H) Leaf-disk senescence assay for salinity stress tolerance in transgenic line (S11). (I) Salinity tolerance index potential of WT and transgenic line (S11). (J) Stress responses of WT and *p68* overexpressing transgenic lines. 14 d-old vermiculite grown seedlings were transferred to without or with NaCl in nutrient solution (0 and 200 mM NaCl). (K) WT and *p68* overexpressing plant (S11) in soil pots supplied without or with 200 mM NaCl solution. Note that the WT plant could not sustain growth under salinity stress. (L) Phenotypic comparison of pods grown in water and stress condition of WT and transgenic line (S11).

To establish the functional significance of the *p68* gene, the complete ORF of the gene was cloned in Hind III site of pCAMBIA1301 (Figure 3A). Pea *p68*-pCAMBIA-1301 construct was overexpressed in tobacco plants by using *Agrobacterium*-mediated transformation. The integration of the transgene was confirmed by PCR (Figure 3B). The stable integration was also confirmed by Southern blot analysis (Figure 3C). Western blot detected ∼68 kDa band in transgenic lines (Figure 3D). The GUS expression was positive for all the three transgenic lines while no GUS expression was observed in WT plant (Figure 3E). The germination efficiency of the seeds was also tested. The seeds of pea *p68* transgenic lines and WT were grown on MS media containing 200 mM NaCl (Figure 3F). In NaCl containing medium, the seeds of pea *p68* transgenic plants started to germinate at 3 d with germination efficiency of more than 70% (data not shown). In contrast, the germination of WT seeds was observed at 9 d on the NaCl-containing medium. The highest germination rates of WT seeds were 35.6% on the NaCl-containing MS medium (data not shown), which was lower than those of pea *p68* transgenic seeds, suggesting the susceptibility of WT plants to 200 mM NaCl. The seedlings of *p68* transgenic S11 and WT were transferred to pots supplemented with 200 mM NaCl, WT plants died after sometime whereas S11 survived and resumed the growth (Figure 3G). Salinity stress tolerance was also tested by leaf disk senescence assay. Leaf disks of *p68* transgenic line S11 and WT were floated separately on 0 (H_2_O only), 100 or 200 mM NaCl for 72 h. The salinity induced damage was reflected in the degree of bleaching observed in the leaf tissue after 72 h. The leaves of WT plants were bleached, whereas, the leaf disks of S11 retained chlorophyll (Figure 3H). The measurement of salinity stress tolerance index of the 200 mM NaCl treated *p68* transgenic line S11 and WT plants was made using the data of plant dry weight and it was noted that the tolerance potential of *p68* transgenic line S11 was 86.11%, whereas, it was only 30.47% in WT plants (Figure 3I). The performance of *p68* transgenic lines in the presence or absence of salt was similar to the WT plants, which revealed the potential of *p68* in salinity stress tolerance (Figure 3J).

The *p68* transgenic S11 line and WT plants were phenotypically similar when grown in absence of NaCl. To assess the effect of high salt (200 mM NaCl) on growth, morphology and development of *p68* overexpressing and WT plants, the three-week old seedlings were grown in the presence of continuous salt (200 mM NaCl). In the presence of NaCl some stress induced symptoms appeared on the transgenic plants but it still grew normally and set viable seeds, whereas the WT plants could not survive under continuous salinity stress (Figure 3K). In response to stress, the *p68* transgenic set healthy pods similar to H_2_O grown WT plants (Figure 3L). The results show that the *p68* overexpressing transgenic lines have better ability to tolerate salinity stress. p68 overexpressing transgenic lines were further analysed for physiological and biochemical parameters to understand the mechanism of salinity stress tolerance in *p68* overexpressing transgenic lines.

### Segregation Ratio and Seedling Survival of Transgenic and WT Plants Under Salinity Stress

To determine whether the salinity tolerance imparted by *p68* is functionally and genetically stable, the homozygous T_2_ progeny was analyzed. Seeds from the T_0_ plants, when plated onto hygromycin containing medium, segregated in 3∶1 ratio (Table S1). The percent seedlings survival of *p68* overexpressing transgenic lines and WT were also observed and it was found that 200 mM NaCl did not affect the seedlings survival and there was no difference when compared with H_2_O grown WT plants (Table S1).

### Effect of Salinity on Germination of Pollens and Seeds of Pea *p68* Transgenic Tobacco Plants

To compare the germination efficiency, first pollens of WT and *p68* transgenic lines were sown on pollen germination medium (PGM) alone or on medium supplemented with either 100 or 200 mM NaCl. Without addition of NaCl, the germination of the pollen of pea *p68* transgenic was similar to that of WT pollen (Figure S2). The germination of the WT pollen was repressed in the medium containing 100 mM NaCl while no germination was observed in 200 mM NaCl (Figure S2 B, C).

### Effect of Salinity on Growth Performance and Yield Transgenic and WT Plants

Significant growth reduction was noted for the WT plants in response to 200 mM NaCl treatment while *p68* transgenic lines resist the adverse effects of stress by maintaining vigorous growth. Growth performance measured in terms of shoot length, root length, leaf area and plant dry weight remained almost similar in *p68* overexpressing transgenic lines and WT plants under 0 mM NaCl (Figure S3). High concentration of salt (200 mM NaCl) significantly reduced the shoot length, root length, leaf area and plant dry weight of WT plants by 51.70, 57.31, 64.10 and 61.96%, in comparison to 0 mM NaCl. However, the decrease was 16.67, 19.55, 18.82 and 17.35% in the case of S9; 14.50, 18.04, 18.22 and 13.31% in the case of S11 and 18.85, 18.01, 16.92 and 15.62% in the case of S26 in comparison to their respective controls (Figure S3). Under salinity stress, transgenic lines maintained yield contributing parameters including time required for flowering, number of pods per plant, seed number per pod and seed weight per pod and therefore set normal seeds (Table S1).

### Ion (Na^+^ and K^+^) Accumulation in Transgenic and WT Plants under Salinity Stress

**Figure 4:**
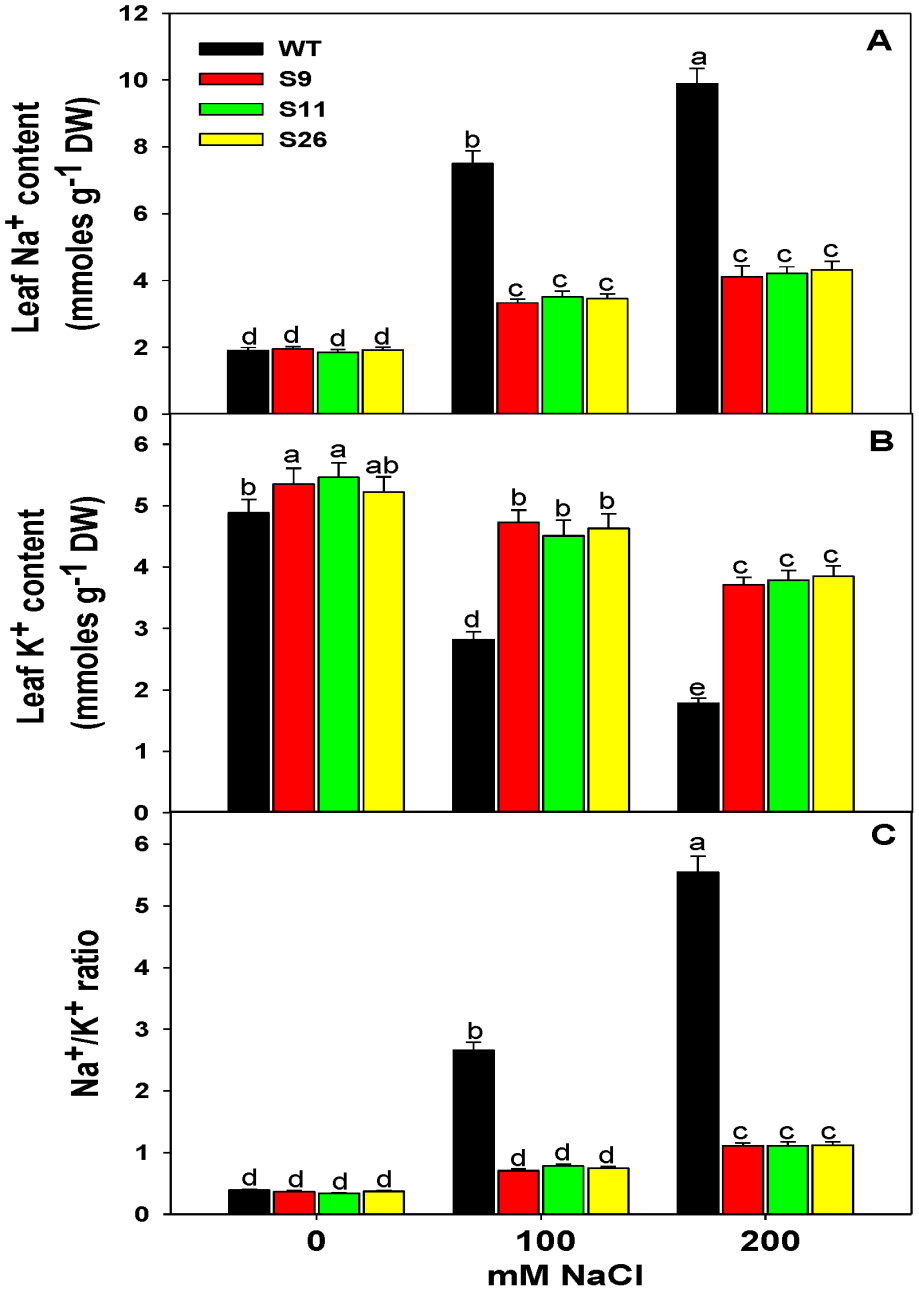
Analysis of ion content in transgenic and WT tobacco plants. (A) Na^+^ content in the leaves of transgenic and WT plants. (B) K^+^ content in the leaves of transgenic and WT plants. (C) Na^+^/K^+^ ratio in the leaves of transgenic and WT plants. The leaves were exposed to 0, 100 or 200 mM NaCl for 3 weeks of salinity treatment. Values are mean ± SE (n = 3). Different letters on the top of bars indicate significant differences at P<0.05 level as determined by Duncan’s multiple range test (DMRT). The results are representative of similar results obtained from two independent experiments.

To observe Na^+^ and K^+^ accumulation, WT and *p68* transgenic plants were exposed to salt stress. No significant difference was observed in the accumulation of Na^+^ in between WT and *p68* overexpressing transgenic lines without NaCl treatment. However, with the increasing salt concentration (100 or 200 mM NaCl), the accumulation of Na^+^ was significantly increased in WT plants while *p68* transformed plants accumulated less Na^+^ (Figure 4A). The pattern of K^+^ accumulation was similar in the leaves of *p68* overexpressing transgenic lines and WT in response to 0 mM NaCl treatment (Figure 4B) but in response to salinity stress, *p68* overexpressing transgenic lines retained more K^+^ compared to the WT plants (Figure 4B). Furthermore, *p68* overexpressing transgenic lines showed lower Na^+^/K^+^ ratio in comparison to WT plants (Figure 4C), which reflect the potential of transgenic lines to tolerate salinity stress.

### Effect of Salinity on Chlorophyll Content, Photosynthesis and Chlorophyll Fluorescence of Transgenic and WT Plants

**Figure 5:**
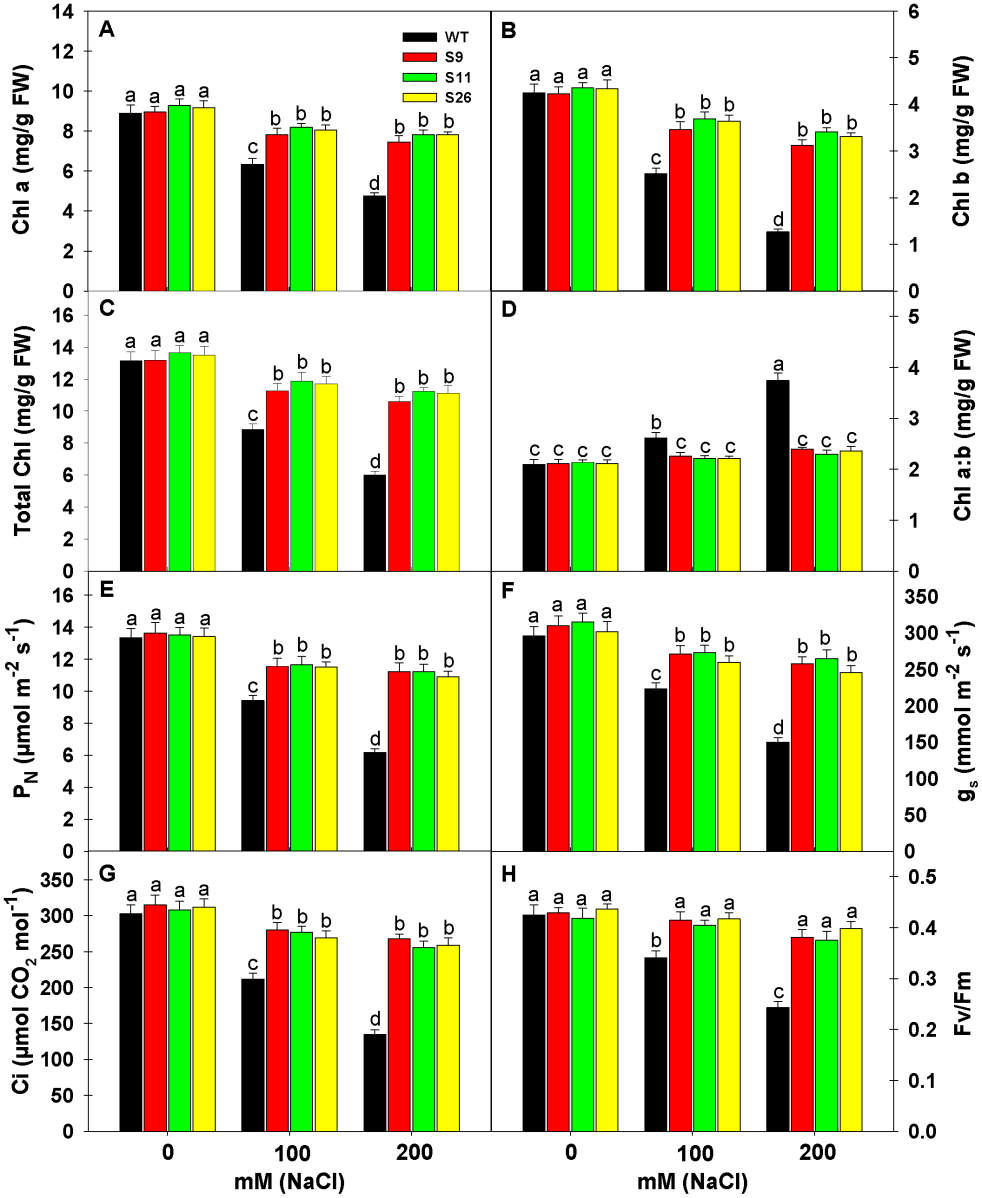
Effects of salinity stress on chlorophyll content, photosynthesis and chlorophyll fluorescence of transgenic and WT tobacco plants. (A) Chlorophyll a content. (B) Chlorophyll b content. (C) Total Chlorophyll content. (D) Ratio of Chlorophyll a and b. (E) Measurement of Net photosynthetic rate (P_N_). (F) Measurement of stomatal conductance (gs). (G) Measurement of internal CO_2_ concentration [(C_i_)]. (H) Measurement of Chlorophyll fluorescence (*F_v_/F_m_*). Data’s were recorded from WT and *p68* overexpressing transgenic tobacco lines after 3 weeks exposure to 0, 100 or 200 mM NaCl treatment. Each value represents mean of three replicates ± SE. Means were compared using ANOVA. Data followed by the same letters are not significantly different at P<0.05 as determined by least significant difference (LSD) test. ^a, b, c^ indicate significant differences at P<0.05 level as determined by Duncan’s multiple range test (DMRT).

To access the effect of salinity on Chlorophyll (Chl), Chl a, Chl b, total Chl and Chl a: b was measured in *p68* overexpressing transgenic lines and WT plants (Figure 5A–D). Salinity stress (100 or 200 mM NaCl) significantly reduced the Chla, Chlb and total Chl in transgenic lines and WT plants but the extent of reduction was higher in WT than *p68* overexpressing transgenic lines. The reduction in Chl a, Chl b and total Chl under 200 mM NaCl in WT was 46.56, 70.12 and 54.18% in comparison to 0 mM NaCl, whereas, the reduction was 16.65, 26.24 and 19.73% in the case of S9; 15.71, 21.61 and 17.59% in the case of S11 and 14.83, 23.73 and 17.69% in the case of S26 in comparison to their respective controls (Figure 5A–C). Under salinity stress the Chl content remained significantly higher in transgenic than WT plants. In WT plants the Chl a:b ratio followed the reverse pattern as of Chl content and significantly increased with the increasing salt concentration, whereas, no significant change was noted in the case of transgenic lines (Figure 5D). It reflects that Chl b was severely affected by salinity stress than Chl a under increasing salt concentration in WT plants than transgenic lines which led to significant increase in Chla: b ratio.

Salinity stress affects the photosynthetic functions at various levels, therefore, the photosynthetic parameters like net photosynthetic rate (P_N_), stomatal conductance (gs) and internal CO_2_ (*Ci*) were measured in *p68* overexpressing transgenic lines (S9, S11 & S26) and WT plants under different salinity levels (0, 100 or 200 mM NaCl) (Figure 5E–G). High level of salinity (200 mM NaCl) significantly reduced the photosynthetic parameters but the extent of reduction was several folds higher in WT plants than transgenic lines. It is interesting to note that *p68* overexpressing transgenic lines maintained higher photosynthesis than WT even under 0 mM NaCl. The reduction in P_N_, gs and *Ci* of WT plants was 53.75, 49.32 and 55.44% under 200 mM NaCl in comparison to their controls, whereas, the decrease in P_N_, gs and *Ci* of S9, S11 and S26 was 17.67, 16.77, 14.92%; 16.94, 15.87, 16.88% and 18.85, 18.54, 16.99%, respectively in comparison to their controls.

Chlorophyll fluorescence measurement is one of the most commonly used parameters to study the ecophysiology of plants under salinity stress. To understand the response of salt stress, we measured maximal efficiency of PSII photochemistry (*F_v_/F_m_*) in *p68* overexpressing transgenic lines and WT plants (Figure 5H). High salinity stress (100 or 200 mM NaCl) significantly reduced the *F_v_/F_m_*, whereas, it remained unaltered in transgenic lines. Statistically insignificant change in *F_v_/F_m_* in *p68* overexpressing transgenic lines reflects that PSII complex did not suffer damage under NaCl stress.

### Less Oxidative Stress in *p68* Transgenic Lines than WT under Salinity Stress

**Figure 6:**
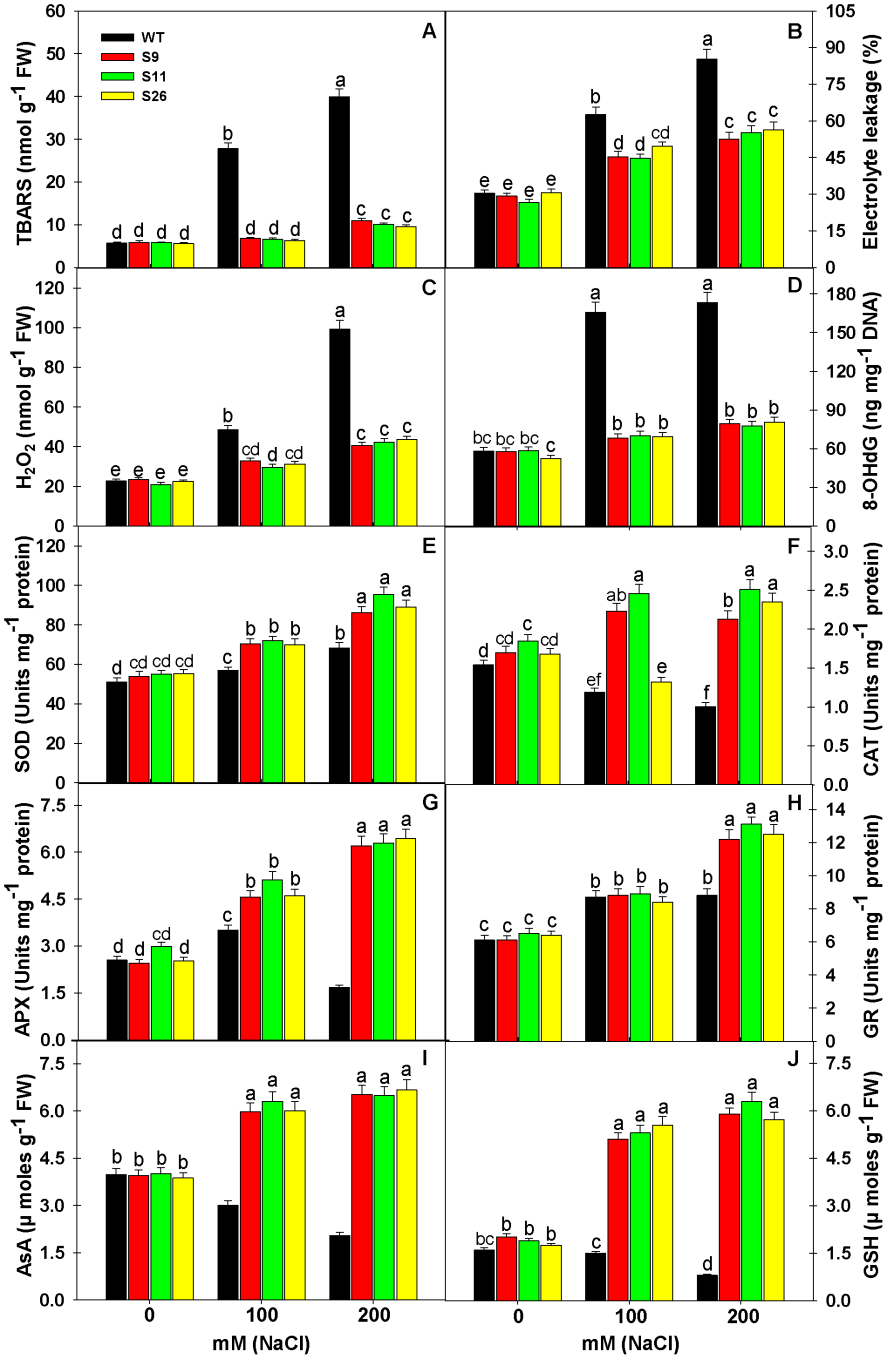
Overexpression of pea *p68* showed less oxidative damage by modulating the ROS machinery under salinity stress. Measurement thiobarbituric acid reactive substance (A), Electrolyte leakage (B), hydrogen peroxide content (C) and oxidative DNA damage in terms of 8-OhdG (D) in the leaves of WT and *p68* overexpressing transgenic tobacco exposed to 0, 100 or 200 mM NaCl after 3 weeks of salinity treatment. Measurement of the activities of superoxide dismutase (E), Catalase (F), ascorbate peroxidase (G) guaiacol peroxidase (H) ascorbate content (I) and glutathione content (J) in the leaves of WT and p68 overexpressing transgenic tobacco exposed to 0, 100 or 200 mM NaCl after 3 weeks of salinity treatment. Values are mean ± SE (n = 3). Different letters on the top of bars indicate significant differences at P<0.05 level as determined by Duncan’s multiple range test (DMRT). The results are representative of similar results obtained from two independent experiments.

Abiotic stresses including salinity cause overproduction of ROS, which leads to oxidative stress in plants. Therefore, the indicators of oxidative stress such as lipid peroxidation (TBARS content), H_2_O_2_ content, electrolyte leakage and oxidative DNA damage were studied in *p68* overexpressing transgenic lines and WT plants (Figure 6A–D). High concentration of salt (100 or 200 mM NaCl) significantly increased the extent of oxidative damage and it was significantly higher in WT as compared to *p68* transgenic lines. The response of oxidative stress parameters reflects non-significant difference in the values of TBARS content, H_2_O_2_ content, electrolyte leakage and oxidative DNA damage (8-OHdG) between *p68* overexpressing transgenic lines and WT plants under no salt i.e. 0 mM NaCl. The increase in TBARS, H_2_O_2_, electrolyte leakage and 8-OHdG under 200 mM NaCl in WT was 600.00, 337.44, 181.03 and 197.59% in comparison to 0 mM NaCl, whereas, the increase was 86.61, 72.76, 79.53 and 37.67% in the case of S9; 74.48, 102.39, 107.08 and 32.48% in the case of S11 and 70.20, 93.78, 84.48 and 53.62% in the case of S26 in comparison to their respective controls (Figure 6A–D). Overall, it is noted that WT plants suffered maximum oxidative damage reflected in terms of peroxidation of lipids, nucleic acid and electrolyte leakage, whereas, *p68* overexpressing transgenic lines experienced less oxidative stress, therefore less oxidative damage.

### Overexpression of *p68* Enhances ROS Scavenging Capacity in *p68* Transgenic Lines

Salinity stress is known to cause ROS induced oxidative damage in plant cells. Therefore, we analyzed the response of enzymatic (SOD, CAT, APX and GR) and non-enzymatic antioxidants (AsA and GSH) in *p68* overexpressing transgenic lines and WT plants under salinity stress. Antioxidant defense machinery protects the plant cells from ROS induced oxidative damage. SOD constitutes the primary step of cellular defense, where SOD dismutates O_2_^• ¯^ to H_2_O_2_ and O_2_. The increase in SOD activity was noted in both *p68* transgenic lines and WT plants but activity of SOD was several folds higher as compared to WT plants. Significant increases in SOD activity in *p68* overexpressing transgenic lines under 200 mM NaCl was 59.92, 73.13, 61.12%, whereas, it was just 33.92% in WT in comparison to their respective controls (Figure 6E). The H_2_O_2_ generated by the action of SOD is restricted through the action of CAT and APX, which reduces H_2_O_2_ to water. In the present study, reverse to SOD activity, the CAT and APX activity decreased significantly in WT plants but CAT and APX activity showed significant increase in *p68* overexpressing transgenic lines under 200 mM NaCl (Figure 6F–G). The decrease in CAT and APX activity was 34.80 and 34.12% in WT plants under 200 mM NaCl. The activity of CAT and APX increased in *p68* overexpressing transgenic lines under 200 mM NaCl by 25.29, 153.06%; 35.68, 111.07% and 39.88, 154.19%, respectively, in comparison to their respective controls (Figure 6F–G). GR catalyzes the NADPH-dependent reduction of oxidized GSSG to the reduced GSH. Here, GR activity showed an increasing trend under salinity stress in both *p68* overexpressing transgenic lines and WT plants but maximum significant increase was seen in *p68* transgenic lines (Figure 6H). Increase in GR activity in *p68* overexpressing transgenic lines under 200 mM NaCl was 100.00, 101.54 and 95.31%, respectively, in comparison to their respective controls, whereas, it was just 44.26% in WT plants. Overall, the upregulation of ROS scavenging antioxidant enzymes in *p68* overexpressing transgenic lines protected the plant cells from the damaging effect of ROS generated by salinity stress.

AsA is the only antioxidant buffer in the apoplast and a key antioxidant that reacts with superoxide and hydroxyl radicals, whereas, GSH is a major non-enzymatic scavenger of ROS. The efficient coordination of AsA and GSH in AsA-GSH cycle can protect the plants from salinity induced oxidative damage. Under control condition (0 mM NaCl), ASC and GSH contents were same in control and transformed plants. However, *p68* overexpression enhances the pool of AsA and GSH in the transgenic lines. The decrease in AsA and GSH content was 48.74 and 50.00% in WT plants under 200 mM NaCl. The content of AsA and GSH increased in *p68* overexpressing transgenic lines under 200 mM NaCl by 65.32, 193.53%; 61.84, 233.33% and 72.35, 226.86%, respectively, in comparison to their respective controls (Figure 6F–G).

## Discussion

Abiotic stress is known to affect the cellular gene-expression that limits crop productivity worldwide. So the molecules that are involved in nucleic acid processing, such as helicases, are expected to be affected in response to stress as well. It is evident that stress triggers the expression of many genes including DEAD-box helicases, which play a crucial role in various abiotic stresses [8], [11]–[12], [14], [17], [24]. In this study, a novel DEAD-box helicase gene (*p68*) was isolated from pea plant which is specifically upregulated in response to salinity. The transcript of pea *p68* is also accumulated at a high level and almost equally in every part (roots, leaves, tendrils and flowers) of the pea plant. This result is consistent with the earlier report of transcript analysis of *AtDRH1* gene expression in *A. thaliana* [19]. Therefore, this gene could be a potential candidate for developing stress-tolerant transgenic plants.

The pea p68 protein contains all conserved domains that are characteristic of the DEAD-box proteins including ‘Q’ and ‘GG’ motifs [25]. The ability of p68 to interact with itself indicated that the oligomerization of p68 may be essential for its proper functioning. The p68 is exclusively localized to the cytoplasm and also seems to surround the nucleus of cell. Similar results were reported for another pea helicase (PDH45) [26]. Previously nuclear localization of some DEAD-box helicases (eg. mammalian *eIF4AIII* and *A. thaliana* UAP56) showed the involvement in nonsense-mediated mRNA decay, mRNA splicing and export [27]–[28]. RNA splicing process involves the association and dissociation of the pre-mRNA with snRNAs, which may be facilitated by RNA helicases. Previous report in animal system has shown that p68 and p72 RNA helicases are the crucial factors required for efficient RNA splicing [29]–[30]. Both p68 and p72 interact with the U1 small nuclear ribonucleoprotein that recognizes the 5′ splice site [31]. The p68 protein, devoid of RNA helicase or ATPase activity also inhibited the dissociation of U1 from 5′ splice site, and downregulation of DDX5 resulted in the accumulation of unspliced RNA [29]. The role of plant p68 in splicing has not been yet reported. However, there may be possibility that the p68 is sustaining the splicing activity during the stress condition and therefore allowing the p68 overexpressing tobacco plant to survive under the salinity stress condition.

Previously a number of studies demonstrated that DEAD-box RNA helicases are involved in regulating many stages of plant development processes including plant morphogenesis, embryogenesis, pollen tube guidance, floral meristems, flowering, plastids and seed development [32]–[34]. In plant the first report of stress induced helicase gene came by cDNA microarray analysis of 1300 *Arabidopsis* genes where the authors reported a DEAD-box helicase gene (accession number AB050574) as a cold stress-inducible gene suggesting a new role of helicases in stress signaling [35]. Later, many plant DEAD-box helicases were identified and found to be activated in response to changing environmental conditions [2], [13]–[14], [36]. Evidence is accumulating that transcript of PDH45 and PDH47 was found to be induced in response to high salt, dehydration and cold stresses [11], [13]–[14], [17], [26]. In barley, a salt-responsive transcript HVD1 is induced under salt stress, cold stress, and ABA treatment [37]. AvDH1 is another DEAD-box helicase gene from the halophyte dogbane plant that also strongly upregulated in response to salinity and low temperature [24]. Under normal growth conditions relatively high level of basal expression of the pea *p68* gene in different plant parts implies its function in growth and/or development processes. Under salt treatment, a single species of pea *p68* mRNA was detected abundantly and constitutively in the tissues examined. This indicated that basic activity of cells might be regulated by pea *p68* under salt stress.

Genome-wide expression analysis of many DEAD-box helicase genes have been identified and suggested that these genes might be stress regulated [38]. Overexpression analysis in different DEAD-box helicases has been shown to provide multiple abiotic stress tolerance in crop plants by regulating different signalling pathways [11]–[12], [17]. For example, overexpression of *PDH45* and *OsSUV3* gene provided salinity stress tolerance in tobacco and rice respectively [12], [17]. LOS4 and RCF1 mutant analysis in *Arabidopsis* was found to play an important role in response to cold and heat stress [8], [15]. Our study showed that overexpression of pea *p68* provides salinity stress tolerance in tobacco.

The reduction in leaf chlorophyll content under abiotic stress has been attributed to the destruction of chlorophyll pigments in various crop plants [3], [39]–[40]. We observed that stress-induced chlorophyll loss was enhanced in WT plant while transgenic lines retained more chlorophyll. This finding has strong correlation with the previous studies in other DEAD-box helicases [12], [41]–[42]. Hence it indicated that overexpression of pea *p68* could have positive effects on the growth and photosynthetic metabolism process. Under stress condition, maintenance of the Na^+^, K^+^ levels and Na^+^/K^+^ ratio are important indicators for plant stress tolerance [43]–[45]. The pea *p68* overexpressing tobacco plants accumulated less Na^+^ and more K^+^ as compared to the WT plants. Higher K^+^ content implies delayed leaf senescence in the transgenic lines. Previously it was reported that lower cytosolic K^+^ content controlled endonuclease and caspase-like proteases activity in the cells causing leaf senescence under stress conditions [40], [46]. Transgenic tobacco plants also extruded more Na^+^ from cells and, as a result, low Na^+^ content was detected which indicated that overexpression of pea *p68* enhances stress tolerance in transgenic plants. In wheat increased K^+^ uptake and Na^+^ extrusion was reported in response to salinity stress [47]. The pea p68 overexpressing transgenic plants are also capable of absorbing more water and diluting the Na^+^ content. Earlier transgenic approaches with transporter proteins and DESD-box helicase (PDH45) showed that lower Na^+^/K^+^ ratio helps plants to respond to salinity stress tolerance [14], [45], [48]. We found lower Na^+^/K^+^ ratio in transgenic lines which suggested overexpression of pea *p68* might restrict the entry of Na^+^ ions into the cells thereby protecting photosynthetic machinery from abiotic stresses.

Stress also leads to the rapid production of ROS including H_2_O_2_ in plant tissues that ultimately cause damages to the cell membrane and other cellular components such as plasma membrane, mitochondria and chloroplasts [14], [40]. Hence, to avoid any stress-induced injuries plant needs to develop efficient mechanism to remove excess ROS from cells. Enzymatic ROS-scavenging and non-enzymatic antioxidants system are such mechanisms in the plant cells that prevent ROS induced oxidative damage [49]–[51]. In the present study, stress-induced high H_2_O_2_ accumulation was noted more in WT plants in comparison to the transgenic lines. Therefore, we speculated that transgenic plants are capable of removing excess H_2_O_2_ from the cells, hence preventing cellular damages. Catalase, ascorbate and peroxidase are the major enzymes that are known to be involved in scavenging of cellular production of H_2_O_2_ [52]–[53]. Interestingly in this study the activity of these enzymes increased in transgenic lines in response to stress treatment. This indicated that overexpressing lines could readily scavenge H_2_O_2_ either decomposing it through increased activity of catalase or by ascorbate through the ascorbate/glutathione cycle. Previously, a number of overexpression studies have shown an increased activity of catalase, ascorbate and peroxidase in response to abiotic stress treatment [14], [54]–[56].

The involvement of DEAD-box helicases in various metabolic processes in plant cells might have general implications. The present study provides new insights into the novel function of p68 DEAD-box protein in conferring salinity stress tolerance in transgenic tobacco plants without affecting yield. The overexpression of stress-induced DEAD-box *p68* can also provide a good example of the exploitation of factors of RNA metabolism pathways including splicing factor for enhanced agricultural production of economically important crops under stress conditions.

## Supporting Information

Figure S1 **Cloning and sequence analysis of** ***p68*** **.**
(TIF)

Figure S2 **Percent pollen germination of WT and transgenic lines under salinity stress.**
(TIF)

Figure S3 **Effects of salinity stress on growth parameter of transgenic and WT tobacco plants.**
(TIF)

Table S1 **Comparison of segregation ratio, plant seedlings survival and various yield parameters of the WT and transgenic plants.**
(DOCX)
