# Supplementary figures and images for: Pea p68, a DEAD-Box Helicase, Provides Salinity Stress Tolerance in Transgenic Tobacco by Reducing Oxidative Stress and Improving Photosynthesis Machinery
Source: PLoS One. 2014 May 30;9(5):e98287. doi: 10.1371/journal.pone.0098287 (PMC4039504; doi:10.1371/journal.pone.0098287)

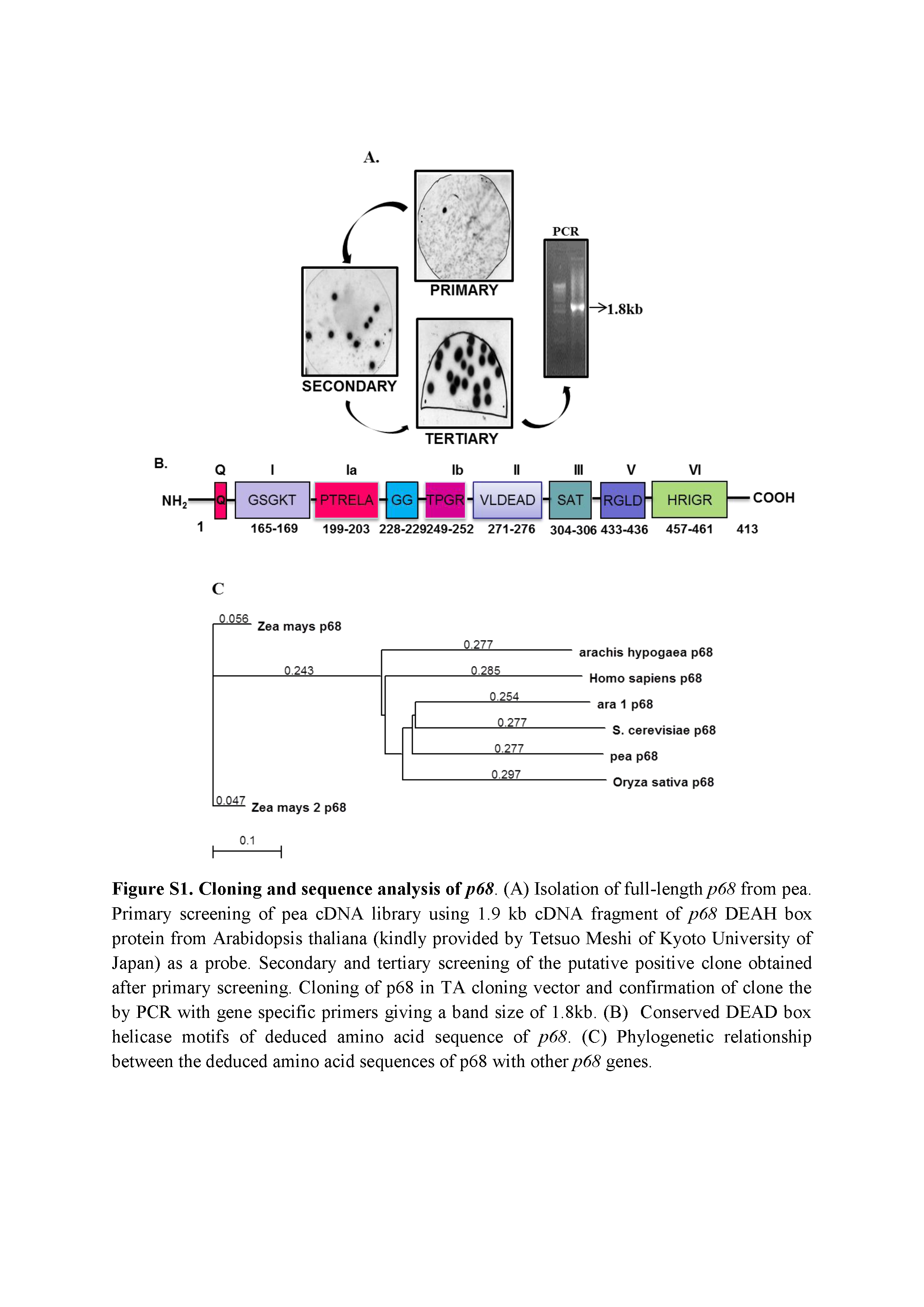

Supplement: Figure S1 — Cloning and sequence analysis of p68 . (TIF) [file pone.0098287.s001.tif]

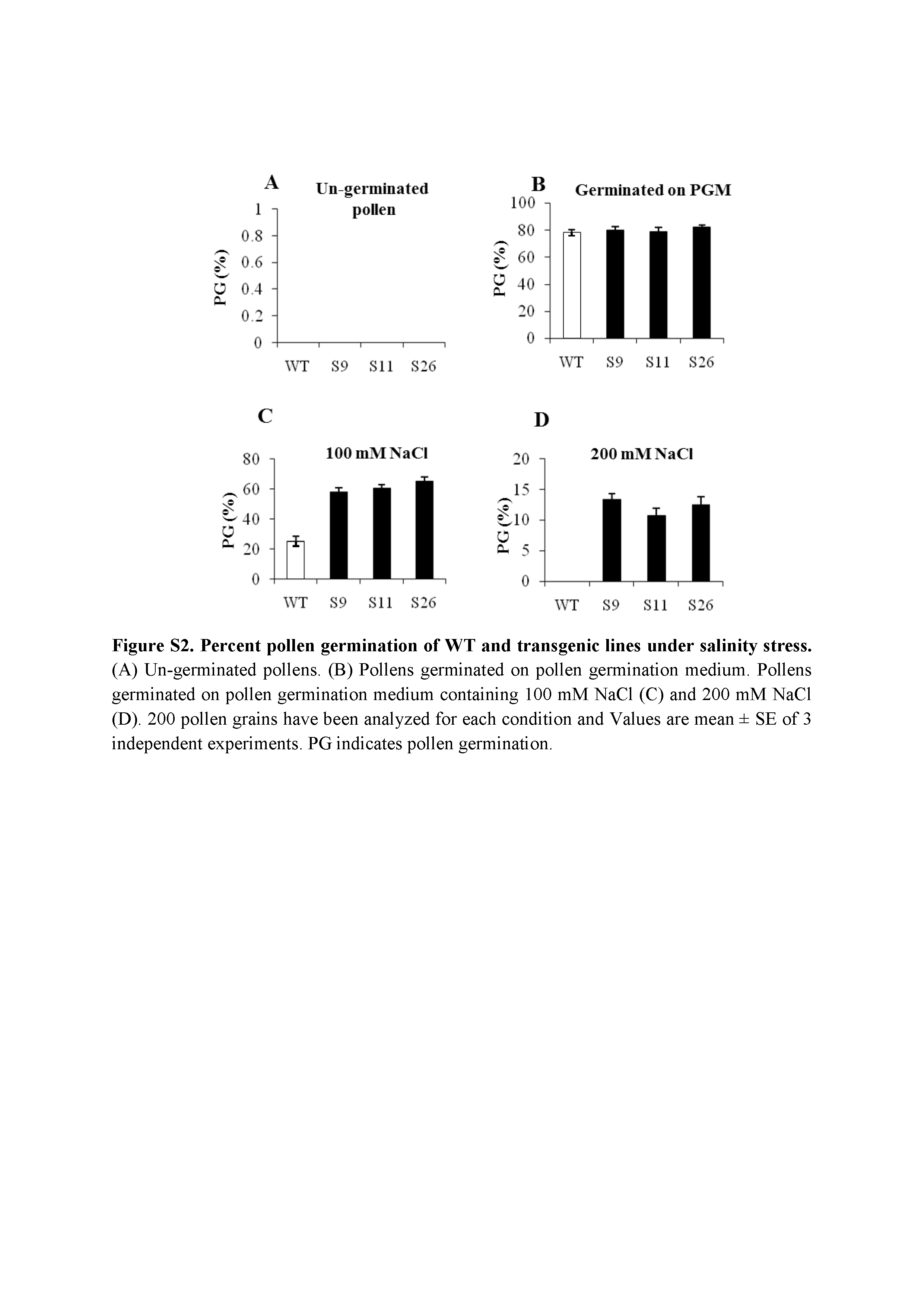

Supplement: Figure S2 — Percent pollen germination of WT and transgenic lines under salinity stress. (TIF) [file pone.0098287.s002.tif]

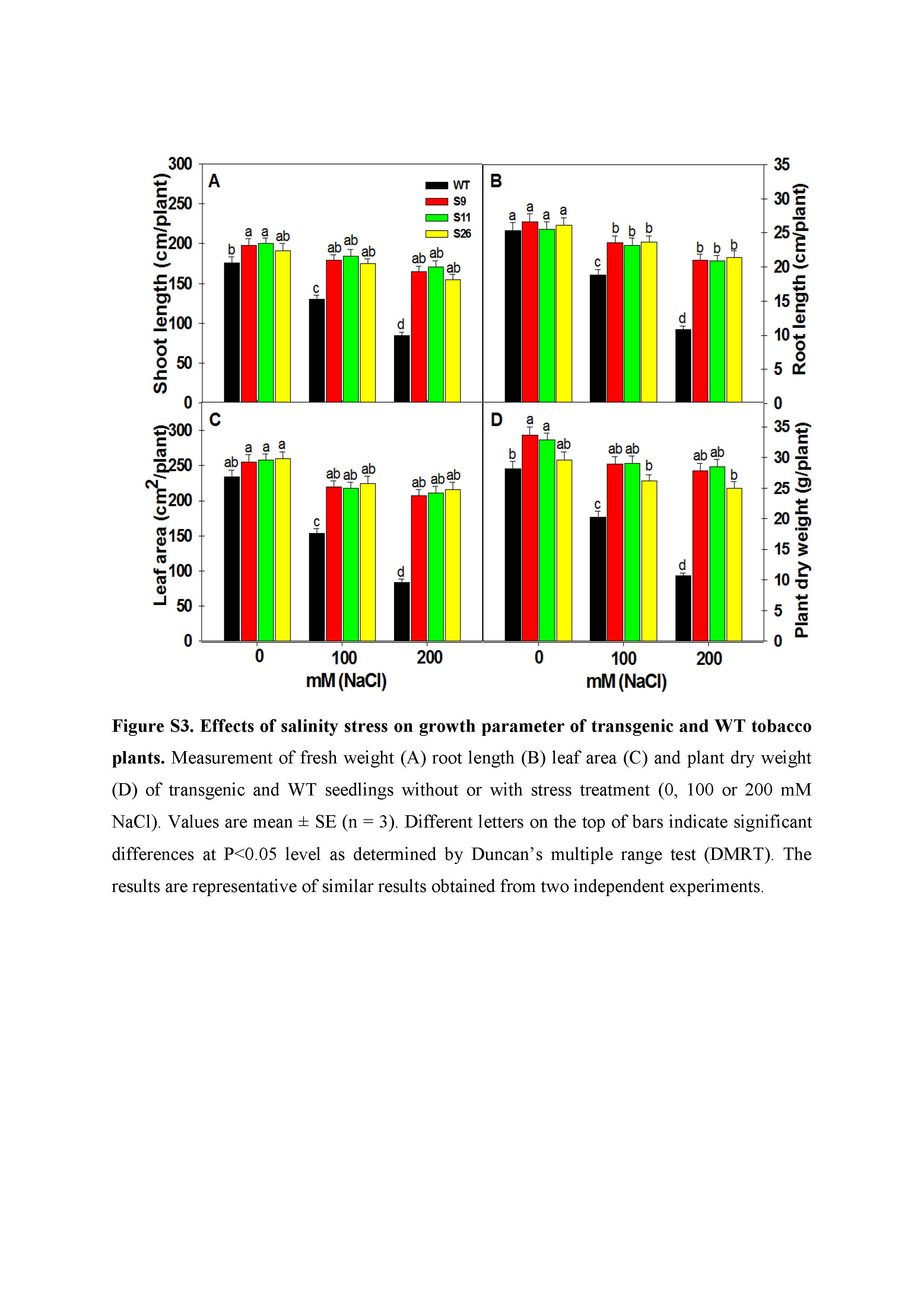

Supplement: Figure S3 — Effects of salinity stress on growth parameter of transgenic and WT tobacco plants. (TIF) [file pone.0098287.s003.tif]
