# Supplementary material for: Pea p68, a DEAD-Box Helicase, Provides Salinity Stress Tolerance in Transgenic Tobacco by Reducing Oxidative Stress and Improving Photosynthesis Machinery
Source: PLoS One. 2014 May 30;9(5):e98287. doi: 10.1371/journal.pone.0098287 (PMC4039504; doi:10.1371/journal.pone.0098287)
Supplement: Table S1 — Comparison of segregation ratio, plant seedlings survival and various yield parameters of the WT and transgenic plants. (DOCX) [file pone.0098287.s004.docx]

**Table S1: Comparison of segregation ratio, plant seedlings survival and various yield parameters of the WT and transgenic plants.**

| Parameter | H_2_O grown  WT | Salt grown tobacco transgenic plants  (200 mM NaCl) | | |
| --- | --- | --- | --- | --- |
|  |  | p68 (S9) | p68 (S11) | p68 (S26) |
| Segregation ratio Hr:Hs [n] €  (T_1_ generation) | 0 | 3:1 [154] | 3:1 [151] | 3.1:1 [158] |
| Percent plant seedlings survival (T_2_ generation) | 95 | 96 | 98 | 97 |
| Time required for flowering (days) (T_2_ generation) | 121±4.35^b^ | 120±2.89^ab^ | 122±4.01^a^ | 118±4.05^b^ |
| Number of pods/plant  (T_2_ generation) | 40±1.55^a^ | 40±1.07^a^ | 42±1.63^a^ | 39±2.01^a^ |
| Seed number per pod  (T_2_ generation) | 1999±53.76^a^ | 2001±55.37^a^ | 2022±64.81^a^ | 1995±58.34^a^ |
| Seed weight/pod (grams)  (T_1_ generation) | 0.179±0.003^b^ | 0.181±0.005^ab^ | 0.188±0.004^a^ | 0.175±0.0003^b^ |

€ Recording made from seeds. Each value represents mean of three replicates ± SE (n=3). Data followed by the same letter in the same row are significantly not different at *P* < 0.05 as determined by Duncan’s multiple range test.
